# Viscoelastic Characterization of Corn Starch Paste: (I) The First Normal Stress Difference of a Cross-Linked Waxy Corn Starch Paste with Sucrose

**DOI:** 10.3390/bioengineering9090465

**Published:** 2022-09-13

**Authors:** Shuxin Huang

**Affiliations:** 1 Department of Engineering Mechanics, Shanghai Jiao Tong University, Shanghai 200240, China; huangshuxin@sjtu.edu.cn; 2 Key Laboratory of Hydrodynamics of the Ministry of Education, Shanghai Jiao Tong University, Shanghai 200240, China

**Keywords:** corn starch paste, viscoelastic property, constitutive equation, power law strain model, linear strain model

## Abstract

Experimental viscoelastic data and the corresponding theoretical analysis of corn starch paste in the past 30 years indicate an evident deficiency of the viscoelastic characterization of the paste. The purposes of the study are to check the capability of a recent model on describing the viscoelasticity of the paste and to improve the viscoelastic analysis. The linear viscoelastic property; the steady shear viscosity and the first normal stress difference (*N*_1_) of a cross-linked waxy corn starch paste mixed with sucrose experimentally reported in 2003 were characterized with a structuralized viscoelastic constitutive equation in the present paper. The structuralized parameter *f* in the equation was obtained using the viscosities in the dynamic and steady shear experiment. Both a power law strain model and a linear strain model were proposed to describe the normal component in the strain matrix. Three kinds of viscoelastic properties of the paste can be described well with the structuralized equation. Both the power law and the linear strain model can yield reasonable calculations of *N*_1_. The maximum deviation of *N*_1_ calculated by two strain models is about 10%. The theoretical model adopted is available for describing the complex viscoelastic behaviors of corn starch paste usually appearing in the processing of corn starch.

## 1. Introduction

Starch is a raw material in food industry, which can be eaten directly as food or as an additive. Corn starch is the cheapest and main source of starch and is used widely in food processing and other industries, such as pharmaceutical. The viscoelastic property of corn starch paste or the mixture of corn starch and other substances, such as polysaccharide gum, has been extensively researched for controlling the quality of foodstuffs, e.g., acceptability, texture, and stability [1,2,3,4,5], as well as for adjusting the technological parameters of processing, e.g., piping, mixing, and extrusion [6].

Many studies on the viscoelastic properties of corn starch paste usually contain two kinds of experimental data, i.e., viscosity and linear viscoelastic property [4,5,6,7,8,9]. The power law model [9,10] or the Herschel–Bulkley model [11,12] is adopted for characterizing the viscosity of the paste, and in some works, the power law is also used to analyze linear viscoelastic data [13]. Ptaszek and Grzesik [14] (2007) once characterized the linear viscoelastic property of the mixture of corn starch and guar gum by using continuous relaxation spectrum. Due to the lack of detailed theoretical characterization on the viscoelasticity of corn starch paste, it is not easy to simulate the flow of corn starch paste during piping, mixing, and extrusion process or to evaluate the quality of the paste on the basis of extensive and quantitative viscoelastic data. The present theoretical deficiencies have two aspects. One is that each of the reported viscoelastic experimental results for a paste is isolated, and the relationship between these data is not researched. The other is that no theory is examined to simultaneously describe all the viscoelastic data together.

The traditional viscoelastic constitutive equation incorporating the shear rate-dependent structural parameter could be available for characterizing and predicting the viscoelastic property of corn starch paste. The recent work of Huang [15,16] provided a case for predicting the viscoelastic property of polysaccharide mucilage of a frond with a structuralized viscoelastic model, and then, Huang [17] (2021) reduced the model to describe the viscoelasticity of loach skin mucus. The shear rate-dependent structural parameters influence the relaxation spectrum of fluid, which can then tune other viscoelastic properties. The model in Huang [17] will be adopted and modified to characterize the viscoelasticity of corn starch paste in the present work.

In order to characterize and predict viscoelastic data of corn starch paste, experimental data of the paste in the past 30 years were collected in the present paper, which are listed in Table 1. Two criteria for collecting the data are adopted. One is that the experimental viscoelastic data of pure corn starch paste should be contained in the published work and the other is that three kinds of data, i.e., frequency sweep data at small strain, steady shear viscosity, and one of the other viscoelastic data at constant temperature and concentration should be included. The idea of including three kinds of data is for predicting one of the data by using the characterization based on the other two data. From Table 1, it can be seen that the power law model and Herschel–Bulkley (HB) model are often adopted in theoretical analysis. The power law model is adopted in most studies due to two facts. One is that the power law model is the simplest model, which can be used easily. The other is the typical shear thinning viscosity of corn starch paste, which is suitable for characterization with the power law model. Moreover, the Burgers model in Table 1 is used to characterize the creep data.

In Table 1, the simplest data is no. 3′s data obtained by Acquarone and Rao, 2003 [18], because the third kind of data provided in no. 3 is steady shear data and all the others contain transient data. For simplicity, the viscoelastic data in [18] is employed in the present theoretical analysis. The objectives of the present study are to check the capability of the model of Huang [17] on describing the viscoelasticity of corn starch paste and to improve the theoretical analysis on the viscoelasticity of the paste.

## 2. Materials and Methods

### 2.1. Corn Starch Paste

The cross-linked waxy corn starch used by Acquarone and Rao [18] is from National Starch and Chemical Co. in Bridgewater, NJ, USA. Fourteen groups of corn starch pastes were prepared in the work of Acquarone and Rao [18], in which the concentration of cross-linked waxy corn starch is 50 g/kg and the concentrations of sucrose are 0, 100, 200, 300, 400, 500, and 600 g/kg. Half of the pastes, i.e., seven groups of 50 g/kg starch pastes with 0, 100, 200, 300, 400, 500, and 600 g/kg sucrose were heated to 85 °C in Rotavapor (Büchi, Switzerland), and the others were heated in cans at a retort temperature 110 °C. More details on the pastes can be found in [18].

Acquarone and Rao [18] only reported two groups of first normal stress difference data. One was for the corn starch paste with 300 g/kg sucrose heated to 85 °C, and the other for the paste with the same sucrose concentration but heated in cans to 110 °C. The characterization on the experimental viscoelastic data for the paste with 300 g/kg sucrose heated to 85 °C was presented in detail here.

### 2.2. Viscoelastic Model

The viscoelastic model adopted in the calculation is the reduced structuralized Meister-type constitutive equation [17,23,24,25], which contains a structural parameter *f* and is written as,

(1) $$\tau =\int_{-\infty }^{\;t}m\left(t-t^{\prime },f\right)\cdot \phi_{1}\left(I_{1},I_{2}\right)\cdot [\delta -C_{t}^{-1}\left(t^{\prime }\right)]dt^{\prime }$$

where *m*(*t*-*t*′, *f*) is the time and shear rate-dependent memory function, *f* reflects the effect of shear rate on the change of structure or relaxation spectrum, *δ* is the unit tensor, ***C***_t_^−1^ is the Finger strain tensor, *t* and *t*′ are the present and the past time, respectively, with unit of s, *ϕ*_1_(*I*_1_, *I*_2_) is a strain-dependent function, *I*_1_ and *I*_2_ are the first invariants of ***C***_t_^−1^ and ***C***_t_, respectively, and ***C***_t_ is the Cauchy strain tensor.

The memory function used in the reduced model [17] is,

(2) $$m=\sum_{i}\frac{g_{i}\cdot f(\dot{\gamma })}{\lambda_{i}}\cdot e^{\left(-\frac{t-t^{\prime }}{\lambda_{i}}\right)}$$

where *λ*_i_ and *g*_i_ are the relaxation times (s) and the relaxation modulus coefficients (Pa), respectively, at low shear rate or at rest. The *ϕ*_1_-function in shear flow for the corn starch paste is [26],

(3) $$\phi_{1}\left(I_{1},I_{2}\right)=\frac{1}{\sqrt{1+\gamma^{2}}}$$

where *γ* is shear strain.

Equation (1) without parameter *f* is the reduced Rivlin–Sawyers (RS) equation without *ϕ*_2_-function term [24,25], and then, if the *ϕ*_1_-function is a single exponential model, Equation (1) becomes the Meister equation [23]. Equation (1) is solved numerically by the Laguerre–Gauss integration method, which is fulfilled via a Fortran code [27].

### 2.3. Characterization of Viscoelastic Property

To characterize the viscoelastic properties of the corn starch paste with the model above, the relaxation spectrum (*λ*_i_, *g*_i_) and the structural parameter *f* should be obtained, in which the spectrum is obtained using the linear viscoelastic data and the parameter *f* is obtained using the shear viscosity. Then, the first normal stress difference, i.e., *N*_1_, will be predicted. The details of the corn starch dispersion viscoelastic experiment with 300 g/kg sucrose heated to 85 °C can be found in Acquarone and Rao [18].

The linear viscoelastic property is vital for characterizing the viscoelastic property of corn starch dispersion [4,5,6,7,8,9], polysaccharide solution [15], and polysaccharide gel [28]. The experimental linear viscoelastic data of the corn starch paste measured at the small strain in the oscillation shear experiment can be fitted by using the following equations,

(4) $$G^{\prime }\left(\omega \right)=\sum_{i}\frac{g_{i}\lambda_{i}^{2}\omega^{2}}{1+\lambda_{i}{}^{2}\omega^{2}}$$

(5) $$G^{\prime \prime }\left(\omega \right)=\sum_{i}\frac{g_{i}\lambda_{i}\omega }{1+\lambda_{i}^{2}\omega^{2}}$$

where *G*′ and *G*″ are the storage and the loss modulus, respectively, and *ω* is the experimental angle frequency. The fitting process of Equations (4) and (5) can be found in Bird, Armstrong, and Hassager [25], which is fulfilled with a solver function in Excel 2003 of Office software of Microsoft Co (Redmond, Washington State, USA).

## 3. Results and Discussion

### 3.1. Linear Viscoelastic Property

**Figure 1:**
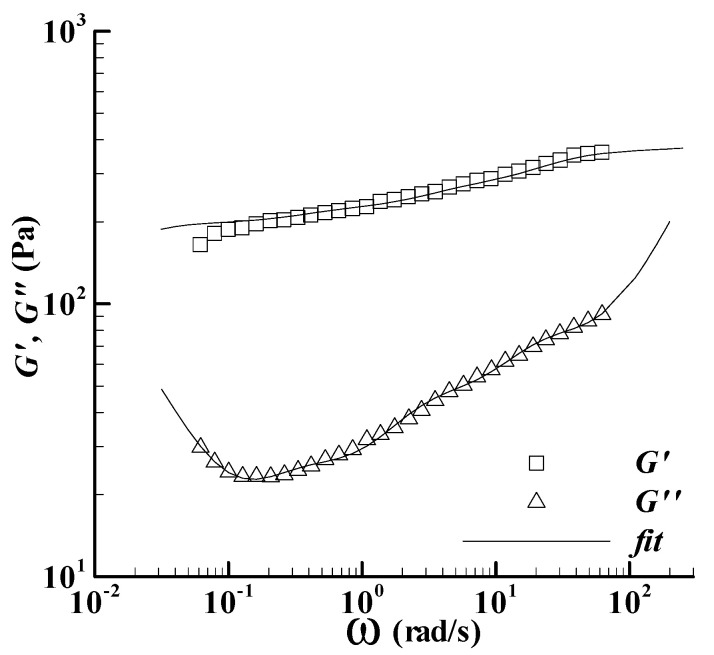
The linear viscoelastic property of the cross-linked waxy corn starch dispersion at 20 °C, which is mixed with 300 g/kg sucrose and heated to 85 °C in the Rotavapor. The symbols are the experiments in Figure 7a of Acquarone and Rao [18], and the lines are the fits here.

The fitting results for the dispersion are shown in Figure 1, together with the experiments of Acquarone and Rao [18]. The fits agree with the experiments. The relaxation spectrum is listed in Table 2.

### 3.2. Viscosity

**Figure 2:**
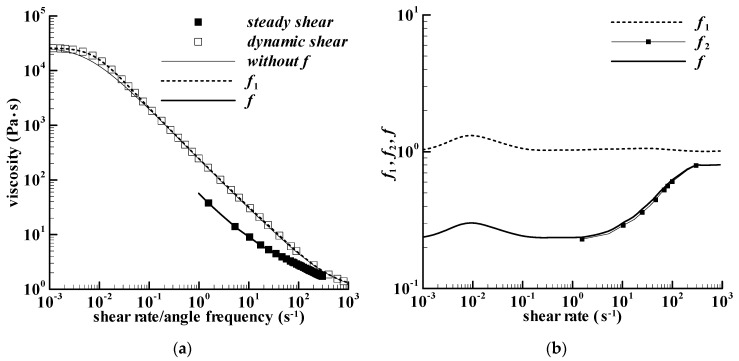
Viscosity of the paste and the calculation parameters used. (**a**) Viscosities of the cross-linked waxy corn starch dispersion at 20 °C, which is mixed with 300 g/kg sucrose and heated to 85 °C in the Rotavapor. The solid square symbol is the experiment in Figure 3a of Acquarone and Rao [18], the square symbol is the calculation using the relaxation spectrum in Table 2, which is regarded as the experiment, and the lines are the calculations; (**b**) The parameters, *f*_1_, *f*_2_, and *f* used in the calculation.

As the relaxation spectrum has been given in Table 2, the magnitude of the complex viscosity, simply called complex viscosity here, can be calculated theoretically in a large range of angle frequencies. Figure 2a shows the calculated complex viscosity, which is denoted by ‘dynamic shear’. If the angle frequency in the complex viscosity is regarded as shear rate, a group of the assumed steady shear viscosity can be obtained. Figure 2a also shows a calculation on viscosity with the viscoelastic model above, i.e., Equations (1)–(3), but not including the *f*-parameter, which is denoted by ‘without f’. It can be seen that the calculation has a slight deviation from the assumed steady shear viscosity in the shear rate range of about 0.001–0.05 s^−1^, and the similar phenomenon can also be seen in the earlier work elsewhere [26]. The reason for the deviation is not clear here, which could be the deficiency of the *ϕ*_1_-function or shear rate effect. The shear rate effect is adopted here to eliminate the deviation by using parameter *f*_1_, which is obtained using the assumed steady shear viscosity over the calculated viscosity without *f*-parameter. The *f*_1_-parameter is shown in Figure 2b, and the calculation on the viscosity by including parameter *f*_1_ has been presented in Figure 2a and denoted by ‘*f*_1_’.

The deviation between the calculated viscosity with *f*_1_-parameter and the experimental steady shear viscosity of Acquarone and Rao [18] is apparent in Figure 2a, which is assumed to be caused by shear rate effect. Then, the *f*_2_-parameter obtained using the experimental viscosity over the assumed steady shear viscosity is employed to eliminate the deviation. Both *f*_1_- and *f*_2_-parameters are related to shear rate effect, and therefore, the parameter *f* = *f*_1_ × *f*_2_ can be adopted here to characterize shear rate effect assumed. Both *f* and *f*_2_ have been given in Figure 2b. The calculation of the steady shear viscosity with *f*-parameter in Figure 2a agrees with the experiment of the corn starch paste with 300 g/kg sucrose heated to 85 °C. Both the *f*_1_- and *f*-values are calculated via a linear interpolation method [26].

The experimental shear viscosity of Acquarone and Rao [18] is obtained by using the triangular-loop shear mode, i.e., the shear rate ramps up from 0 to 300 s^−1^ and then down to 0 s^−1^ in 6 min. The viscosity curve in the up region of the shear rate is different from that in the down region. Acquarone and Rao [18] used the down-viscosity curve as the steady shear viscosity in their original Figure 5 for the paste heated to 85 °C, which is acceptable and also adopted in the present paper. In the earlier works of Rao et al., 1997 [10], the authors also used the down-viscosity curve as the steady shear viscosity for cross-linked waxy corn starch. In the work of Tattiyakul and Rao [8], a shear mode with three triangular-loops was reported, and the maximum deviation of the shear stress at the maximum shear rate for the three measurements is about 6.5% for cross-linked waxy corn starch, which also indicates that the down-viscosity curve can be regarded as the steady shear viscosity. In the other group’s work [7], a shear mode with two triangular-loops was reported for cross-linked waxy corn starch, and the viscosity in the second triangular-loop agrees well with the down-viscosity curve in the first triangular-loop. Therefore, the experimental steady shear viscosity of Acquarone and Rao [18] is reasonable.

### 3.3. First Normal Stress Difference

#### 3.3.1. Prediction

**Figure 3:**
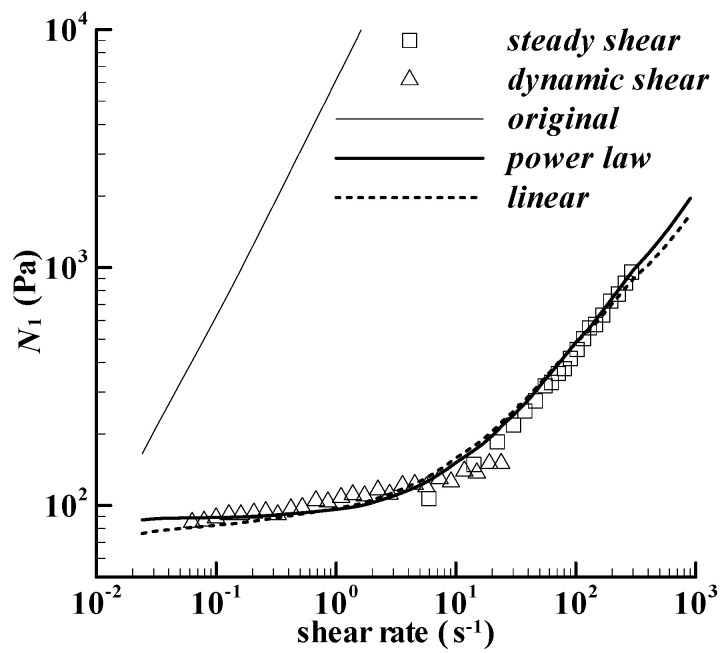
First normal stress difference of the cross-linked waxy corn starch dispersion at 20 °C, which is mixed with 300 g/kg sucrose and heated to 85 °C in the Rotavapor. Symbols are the experiments in Figure 9a of Acquarone and Rao [18], and lines are calculations. The ‘original’, ‘power law’, and ‘linear’ are the calculations using Equations (6)–(8), respectively.

Figure 3 shows the calculation of *N*_1_ with Equation (1), denoted by ‘original’, together with the experiments of Acquarone and Rao [18]. The difference between the calculation and the experiment is significant. Acquarone and Rao [18] obtained the experimental *N*_1_ data by using both the steady shear and dynamic shear experimental mode, which should be reliable for the crosslinked waxy corn starch dispersion mixed with 300 g/kg sucrose. As we know, the elasticity of corn starch paste is weaker than that of polymer melt, which indicates that the deformation of the paste in steady shear flow could not obey the Finger strain. The Finger strain or the Meister-type model is often used to characterize the viscoelastic property of highly elastic polymer solution or polymer melt [23,29]. Therefore, the strain equation used for calculating *N*_1_ should be reconsidered.

#### 3.3.2. Characterization

The original strain component without including one on the diagonal in the Finger strain tensor in simple shear flow [23,29] is,

(6) $$S_{11}(\gamma )=\gamma^{2}$$

In order to characterizing the *N*_1_ of the paste, the following power law normal strain component without including one is assumed,

(7) $$S_{11}(\gamma )=k\gamma^{n}$$

where *k* and *n* are the parameters for describing the special strain component experienced in the deformation of the corn starch paste.

Figure 3 includes the fit on the *N*_1_ with the power law model, i.e., Equation (7), and the calculation agrees well with the experiment for the paste heated to 85 °C. The values of the parameters, *k* and *n*, fitted for the calculation are listed in Table 3. Another check on the power law model is to describe the *N*_1_ for the paste heated to 110 °C, which was also obtained experimentally by Acquarone and Rao [18]. The corresponding parameters for the paste at 110 °C are also listed in Table 3. The *n* values in the two calculations approach 1, which promotes another assumption that *n* = 1 could be available. Thus, the power law model can be written as the following linear model,

(8) $$S_{11}(\gamma )=k\gamma$$

Table 3 lists the values of *k* in Equation (8) for the two pastes heated to 85 °C and 110 °C.

**Figure 4:**
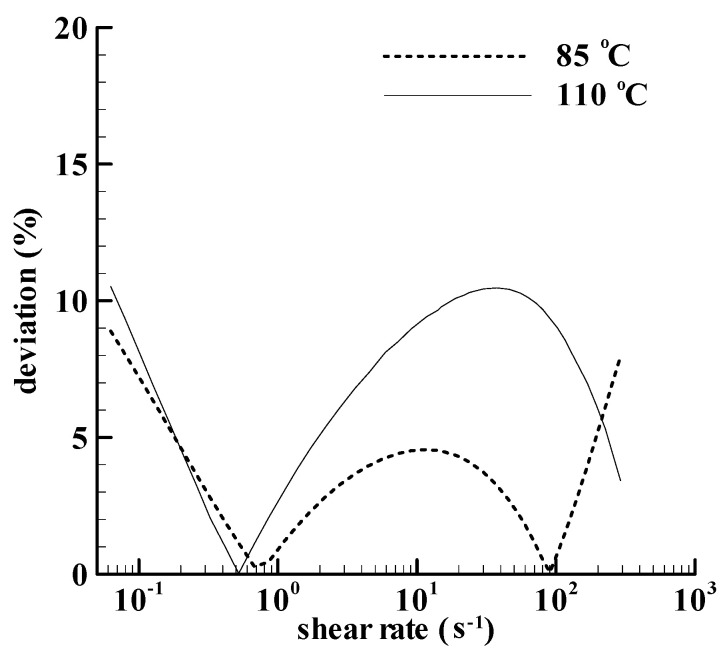
The deviations between the *N*_1_ calculations with the power law strain model and those with the linear strain model for two kinds of corn starch dispersions. One is heated to 85 °C in the Rotavapor, and the other is in cans at a retort temperature of 110 °C.

The calculated *N*_1_ with the linear model in Figure 3 is also acceptable. The maximum deviation between the power law strain model and the linear strain model in Figure 4 for the paste heated to 85 °C is lower than 10%. The definition of the deviation is (|linear calculation—power-law calculation|/power-law calculation) × 100%. Another deviation for the paste heated to 110 °C is also shown in Figure 4, and the maximum deviation is slightly higher than 10%. The linear strain model should be reasonable for the two pastes in [18].

Therefore, the constitutive equation used for characterizing the viscoelastic property of the corn starch paste can be written as,

(9) $$\tau =\int_{-\infty }^{\;t}m\left(t-t^{\prime },f\right)\cdot \phi_{1}\left(I_{1},I_{2}\right)\cdot [\delta -S_{t}\left(t^{\prime }\right)]\cdot dt^{\prime }$$

where *S*_t_(*t*′) is a strain tensor. In shear flow, ***S***_t_(*t*′) is calculated using the following equation,

(10) $$S_{t}\left(t^{\prime }\right)=\left(\begin{array}{ccc} 1+S_{11}\left(\gamma \right) & \gamma & 0\\ \gamma & 1 & 0\\ 0 & 0 & 1 \end{array}\right)$$

where *S*_11_(*γ*) is written as Equation (7) or (8) for the corn starch paste. If *S*_11_ = *γ*^2^, Equation (10) is equal to ***C***_t_^−1^. If Equation (8) is adopted, the strain component is linear for the calculation of the *N*_1_, the same linear dependence on strain as used for calculating the shear viscosity. The linear dependence on strain in the calculation of *N*_1_ for the two corn starch pastes should be checked further for the other corn starch paste or the other macromolecular fluid.

As said by Acquarone and Rao [18] and Tecante and Doublier [7], cross-linked waxy corn starch contains an additional chemical bond, which can strengthen the native starch. The elasticity or the first normal stress difference of cross-linked waxy corn starch dispersion should be stronger than that of native starch, and therefore, the power law strain model should be suitable for common corn starch paste.

## 4. Conclusions

A structuralized Meister-type constitutive equation was modified here to well describe three kinds of viscoelastic properties of a crosslinked waxy corn starch paste with sucrose simultaneously, which are the linear viscoelastic property, the steady shear viscosity, and the first normal stress difference. The modified model is composed of Equations (2), (3), (7), (9), and (10) or of Equations (2), (3), (8)–(10) in shear flow. Both the power-law strain model and the linear strain model are suitable for characterizing the *N*_1_ of the corn starch paste.

## Figures and Tables

**Table 1:** Viscoelastic properties of corn starch pastes in some literature.

No.	Material *	Experiment **	Theory ***	Ref.
1	CWCS	*G*′-*ω*, *G*″-*ω*, *η*-$\dot{\gamma }$, *η*-*t*	power law, HB	[10]
2	CWCS	*G*′-*ω*, *G*″-*ω*, *η*-$\dot{\gamma }$, *η*-*t*	power law	[9]
3	CWCS, sucrose	*G*′-*ω*, *G*″-*ω*, *η*-$\dot{\gamma }$, *N*_1_-$\dot{\gamma }$	power law	[18]
4	WCS, guar, xanthan	*G*′-*ω*, *G*″-*ω*, *η*-$\dot{\gamma }$, multi *η*-*t*	power law	[19]
5	WCS, xanthan, sucrose	*G*′-*ω*, *G*″-*ω*, *η*-$\dot{\gamma }$, *η*-*t*, multi *η*-*t*	power law	[20]
6	NCS, WCS	*G*′-*ω*, *G*″-*ω*, *η*-$\dot{\gamma }$, *η*-*t*, multi *η*-*t*	power law	[13]
7	NCS, oil, water	*G*′-*ω*, *G*″-*ω*, *η*-$\dot{\gamma }$, *J*-*t*	HB, Burgers	[21]
8	NCS, HACS	date *G*′-*ω*, *G*″-*ω*, *η*-$\dot{\gamma }$, *r*-*t*	power law	[22]
9	NCS, cellulose, polydextrose	*G*′-*ω*, *G*″-*ω*, *η*-$\dot{\gamma }$, multi *η*-*t*	HB	[12]

* The material at least includes pure corn starch paste with experimental viscoelasticities reported. The first C in CWCS is ‘cross-linked’, W is ‘waxy’, CS is ‘corn starch’ or ‘maize starch’, N is ‘normal’, HA is ‘high amylose’. ** The experiment at least includes frequency sweep, steady shear viscosity, and one of the other viscoelastic properties at constant temperature and concentration. *G*′, storage modulus; *G*″, loss modulus; *ω*, angular frequency; *η*, shear viscosity; $\dot{\gamma }$, shear rate; *t*, time; *N*_1_, first normal stress difference; *J*, creep compliance; *γ*, strain; multi *η*-*t* means the viscosity-time curve in the multi-step rate experiment. *** HB is the Herschel–Bulkley model.

**Table 2:** The relaxation spectrum of the cross-linked waxy corn starch dispersion with 300 g/kg sucrose at 20 °C, which is heated to 85 °C.

i	*λ*_i_ (s)	*g*_i_ (Pa)
1	9.903 × 10^−5^	9.560 × 10^3^
2	4.146 × 10^−2^	8.914 × 10^1^
3	2.977 × 10^−1^	5.166 × 10^1^
4	2.662 × 10^0^	2.782 × 10^1^
5	1.303 × 10^2^	1.990 × 10^2^

**Table 3:** Parameters in the strain models for the normal component of the strain matrix.

Sample	Equation (7)		Equation (8)
	*k*	*n*	*k*
85 °C	2.074	0.945	1.733
110 °C	1.701	1.053	2.024

## Data Availability

The data that supports the findings of this study are available within the article.
